# The Impact of Nutshell Biochar on the Environment as an Alternative Fuel or as a Soil Amendment

**DOI:** 10.3390/ma16052074

**Published:** 2023-03-03

**Authors:** Lukáš Jeníček, Barbora Tunklová, Jan Malaťák, Jan Velebil, Jitka Malaťáková, Michal Neškudla, František Hnilička

**Affiliations:** 1 Faculty of Engineering, Czech University of Life Sciences Prague, Kamýcká 129, 165 00 Prague, Czech Republic; 2 Faculty of Agrobiology, Food and Natural Resources, Czech University of Life Sciences Prague, Kamýcká 129, 165 00 Prague, Czech Republic; 3 Faculty of Economics and Management, Czech University of Life Sciences Prague, Kamýcká 129, 165 00 Prague, Czech Republic

**Keywords:** biomass, biofuel, biochar, calorific value, nut shells, phytotoxicity

## Abstract

Walnut, pistachio, and peanut nutshells were treated by pyrolysis to biochar and analyzed for their possible usage as fuels or soil fertilizers. All the samples were pyrolyzed to five different temperatures, i.e., 250 °C, 300 °C, 350 °C, 450 °C, and 550 °C. Proximate and elemental analyses were carried out for all the samples, as well as calorific value and stoichiometric analysis. For sample usage as a soil amendment, phytotoxicity testing was performed and the content of phenolics, flavonoids, tannin, juglone, and antioxidant activity were determined. To characterize the chemical composition of walnut, pistachio, and peanut shells, lignin, cellulose, holocellulose, hemicellulose, and extractives were determined. As a result, it was found that walnut shells and pistachio shells are best pyrolyzed at the temperature of 300 °C and peanut shells at the temperature of 550 °C for their use as alternative fuels. The highest measured net calorific value was in pistachio shells, which were biochar pyrolyzed at 550 °C, of 31.35 MJ kg^−1^. On the other hand, walnut biochar pyrolyzed at 550 °C had the highest ash share of 10.12% wt. For their use as soil fertilizers, peanut shells were the most suitable when pyrolyzed at 300 °C, walnut shells at 300 and 350 °C, and pistachio shells at 350 °C.

## 1. Introduction

To protect the environment and sustain economic growth, renewable energy technologies such as solar, hydro, wind, geothermal, and biomass are gaining attention [1]. Biomass is a biodegradable and organic material derived from renewable plant, algae, or animal materials that can be used as bioenergy resources to replace fossil fuels [2]. Biomass energy accounts for the largest share of renewable energy sources available today, providing five times more energy than is contributed by wind and solar sources globally [3]. Biomass derived from agricultural or forestry by-products such as sawdust [4], bagasse [5], coffee husk [6], sugarcane skin [7], and groundnut shell [5] are utilized in large quantities as solid fuels for domestic cooking and heating in developing countries [8].

The United States is the market leader of nut production, increasing its output significantly since 1980 [9]. In 2013, the U.S. production of almonds reached 950 thousand tons, having doubled since 2006. This accounts for over 80% of the global production [10]. Both markets maintained a 4% annual growth rate in 2014, with the U.S. almond sector producing more than 588 thousand tons of shells and the walnut industry producing more than 470 thousand tons of shells [11].

Due to its low bulk density, uneven size, and poor combustion efficiency, nutshell waste should generally not be burned directly [12]. Because of this, additional toxic gas emissions are produced during combustion, and transportation and storage are very expensive. Therefore, residual biomass is converted to biochar with improved physical and chemical characteristics, higher calorific value, higher durability, and lower pollution emissions [4,6,7].

Today, biochar is mainly produced from forestry [13] and agricultural waste [14,15], solid waste [16,17,18], and organic matter [19,20], such as in wood [21], straw, rice husks, and walnut shells [22]. Biochar is recognized as a versatile substance; modified biochar can be utilized for carbon sequestration depending on the surface characteristics, structure, and composition of distinct biochar [23], low-cost adsorbents [24,25,26], as a soil enhancer [27], or as a catalyst carrier [28]. The physicochemical properties of raw materials such as mineral content [29], organic carbon [30], and surface characteristics [31] influence the final characteristics of the produced biochar.

One of the options to convert organic material to biochar is to use the method of pyrolysis. Low-temperature pyrolysis, up to 300–350 °C, is known as torrefaction [32]. Studies show that the calorific value of biomass feedstocks improves significantly when the biomass is pyrolyzed [33].

The benefits of pyrolysis, a pre-treatment method that involves slow heating in an inert or decreased environment, are numerous. The bulk and energy density of biomass can be enhanced by lowering its moisture content [34,35]; lowering the oxygen–carbon ratio (O/C), increasing the calorific value [36,37,38,39]; increasing the hydrophobicity to make it easier to transport and store [40]; and enhancing the ignitability, reactivity, and grindability to reduce energy consumption during grinding and subsequent pulverization [41]. Uslu [42] reported that torrefaction had higher process efficiency than pyrolysis or pellet production, bringing it to a crucial turning point that may lower the price of biofuels and subsequently of renewable energy.

By enhancing characteristics before transit, torrefaction significantly reduces the costs of transporting and storing the biomass needed for large-scale biorefineries to produce energy products [34].

Detailed information regarding the original feedstock and necessary pyrolyzed biomass material qualities must be explored in order to optimize the thermal processing parameters (i.e., temperature and residence time) [43,44]. Studies on pyrolysis are essential to determine the best conditions for manufacturing because the final product depends on residence time but primarily on the temperature used [45]. Chiou [46] confirmed that temperature had a greater impact on the mass and energy production during the torrefaction of fruit and nut shells than time.

Almond, walnut, and coconut are the three nutshell varieties that are most researched. Pyrolyzed almond and walnut shells have a high solid and energy output, increasing their calorific value by eliminating moisture and volatile components [47] while keeping a low sulfur content [46], making them an attractive source for US power plants [45]. By pyrolyzing walnut shells, biochar and bio-oil are frequently produced [48]. The output of biochar produced from walnut shells and distillation waste could be successfully increased by performing torrefaction in addition to pyrolysis [45].

Almond and walnut shells could be pyrolyzed as an alternative to their actual primary purpose. Frequently, walnut shells are used as abrasives for cleaning, and almond shells are utilized as bedding for animals [49]. The torrefied shells can be processed further using pyrolysis or gasification, or they can be utilized as alternatives to regular coal [50,51].

Torrefaction of coconut shells was concluded to be optimal at 275 °C and 30 min of residency, which significantly increased their calorific value to 34.37 MJ kg^−1^ [2,52,53]. For palm kernel shells, pine kernel shells, and almond shells, Arnsfeld stated that the desired results were reached through torrefaction at approximately 300 °C [38].

Briquettes made from biomass feedstock can also be densified, which may be a simple solution to the energy needs for cooking and heating in rural regions [54]. Cooking with reduced GHG (greenhouse gas) emissions was demonstrated when fuel briquette of a mixture of cashew nutshell (65%), areca nutshell (25%), and cassava flour (10%) was compressed at the speed of 90 rpm [55]. The results conclude that good-quality briquettes can be produced from blends of areca nutshells and simarouba seed shells [56]. In Europe, half of the renewable energy requirements are satisfied using processed and densified forest products and agricultural residues [57].

Secondary markets for nutshells are not yet well developed. Processing companies in the US claim to have paid USD 11–27 per metric ton in disposal costs to dispose of extra shells. The global demand for shelled nuts increases every year [10], producing more shells that need to be disposed. The goal of this paper was to assess the alternative usage of pyrolyzed nut shells as fuel or soil supplements. The hypotheses are that pyrolyzed nut shells can serve as good alternatives to fossil fuels and that pyrolysis of nut shells can reduce their phytotoxicity so that biochar from nut shells can be used as a soil amendment.

## 2. Materials and Methods

Three different varieties of shells, including walnut, pistachio, and peanut shells, were utilized for the study. 

The English walnut (*Juglans regia* L.) produces the fruit known as the walnut, which has an edible kernel and a tough, woody shell. Walnut shells are usually found in two-segment and rarely also in three- or four-segment structures. The brown seed coat serves as a protective layer, full of antioxidants, and is located between the hard shell and kernel. Archaeological findings in France indicate that the walnut has been cultivated since the Neolithic period. The walnut harvesting season is from August to September [58].

The pistachio is the fruit of the (*Pistacia vera* L.) tree. It also consists of an edible kernel and a hard shell. The seed has a light green flesh with a particular flavor and a mauve skin hue. The fruit ripens when the shell suddenly cracks partially open and turns from green to an autumnal yellow or red [59]. The original geographical spread is from Central Asia and the Middle East, though today’s largest producers are Iran, USA, Turkey, and China [60]. 

The peanut is the nut of the groundnut plant (*Arachis hypogaea* L.) of the *Fabaceae* family. Unlike the others, peanut is a legume. The peanut’s shell is mainly made up of a mesocarp with numerous large veins running through its length [61]. Peanuts originated in South America about six thousand years ago [62].

All the samples were collected by several households over a period of three months and then milled on a Retch SM 100 miller to a particle size of 1 mm. After that, all samples were pyrolyzed by LECO TGA 701 (LECO Corporation, St. Joseph, MI, USA) to 5 different temperatures of 250 °C, 300 °C, 350 °C, 450 °C, and 550 °C. Pyrolysis at lower temperatures below 350 °C is also known as torrefaction. For the purposes of this research, the term pyrolysis is used, as temperatures above 350 °C are also part of the research. All the samples were heated under same conditions, in an inert atmosphere, at a speed of 20 °C·min^−1^ and held here for an additional 30 min at the desired temperature (Table 1).

### 2.1. Proximate and Ultimate Analysis

Each sample underwent proximate and ultimate analysis in order to comprehend the ingredients and material characteristics. Measurements of the elemental composition, moisture content, and ash content were taken in the preliminary analysis. The second phase involved measuring the calorific value.

A LECO TGA 701 thermogravimetric analyzer was used to measure the contents of moisture and ash. One gram of each sample was dried at 105 °C to a constant weight to calculate the moisture content. One gram of each sample was heated to a constant weight at a high oxygen concentration of up to 550 °C to quantify the amount of ash present.

The LECO instrumental biomass combustion method was used to examine the chemical compositions using a LECO CHN628+S elemental analyzer (LECO Corporation, St. Joseph, MI, USA). Each 0.1 g sample was burned in oxygen at 950 °C to measure the amounts of C, H, and N. The amount of oxygen was determined as a deviation from 100% in a dry state. Ethylenediaminetetraacetic acid, rice flour, and oatmeal were used to calibrate the LECO elemental analyzer on a regular basis.

One gram of each sample was put in a stainless-steel crucible and secured in a cylinder while the pressure was increased to 3 MPa and a reference temperature of 28 °C in order to calculate the calorific value. The substance was then ignited with cotton thread within a LECO AC600 isoperbolic calorimeter (LECO Corporation, St. Joseph, MI, USA). Benzoic acid was used to calibrate the instrument. All results of proximate and ultimate analysis were reported on a dry basis.

### 2.2. Stoichiometric Analysis

To better understand the functioning of combustion processes and to compare theoretical and actual combustion results for their subsequent use in power plants, stoichiometric calculations are necessary. Calculations based on stoichiometry are also useful for comparing outcomes for various materials [63]. The reference oxygen content and reference oxygen amount were set at 10% and 10% vol, respectively, in accordance with the technical criteria for biomass combustion [64].

These calculations establish the following:

Calorific value was calculated by utilizing the outcomes of the proximate and final analyses of each sample, based on the material’s moisture content. The calorific value can be calculated as gross calorific value *Q_s_* (kJ kg^−1^) and net calorific value *Q_i_* (kJ kg^−1^) [64]. The statistics on net calorific value are utilized in this paper;

The theoretical amount of oxygen required for complete combustion of *O*_2,*min*_ (m^3^ kg^−1^) was derived using the following equation:

(1) $$O_{2,min}=V_{m}\left(O_{2}\right)\cdot \left(\frac{C}{M\left(C\right)}+\frac{H}{M\left(2\cdot H_{2}\right)}+\frac{S}{M\left(S\right)}-\frac{O}{M\left(O_{2}\right)}\right)$$

where *C*, *H*, *S*, and *O* are the contents of carbon, hydrogen, sulfur, and oxygen in the sample (% wt.), *V_m_*(*O*_2_) = 22.39 m^3^ kmol^−1^ is the molar volume of gaseous oxygen under normal conditions, and *M*(*X*) (kg kmol^−1^) is the molar mass of the hypothetical elements that merge with *O*_2_;

The theoretical amount of dry combustion air *L_min_* (m^3^ kg^−1^) was derived using the following equation:

(2) $$L_{min}=O_{2,min}\cdot \frac{100}{C_{atm}\left(O_{2}\right)}$$

where *C_atm_*(*O*_2_) = 23.20% wt. is the mass concentration of oxygen in the air; 

The theoretical dry flue gas volume *v_fg_*_,*min*_ (kg kg^−1^) was derived using the following equation:

(3) $$v_{fg,min}=\frac{V_{m}\left(CO_{2}\right)}{M\left(C\right)}\cdot C+\frac{V_{m}\left(SO_{2}\right)}{M\left(S\right)}\cdot S+\frac{V_{m}\left(N_{2}\right)}{M\left(N_{2}\right)}\cdot N+\frac{C_{atm}\left(N_{2}\right)}{100}\cdot L_{min}$$

where *V_m_*(*X*) (kg kmol^−1^) is the molar mass of the flue gas components and *C_atm_*(*N*_2_) = 75.474% wt. is the concentration of *N*_2_ in the air;

The theoretical amount of *CO*_2,*max*_ (kg kg^−1^) emission concentration was derived using the following equation:

(4) $$CO_{2,max}=\frac{M\left(C\right)\cdot C}{V_{m}\left(CO_{2}\right)\cdot v_{fg,min}}$$

The volumetric amount of combustion products was determined using the following equations:

(5) $$v\left(CO_{2}\right)=\frac{V_{m}\left(CO_{2}\right)}{M\left(C\right)}\cdot C+\frac{C_{atm}\left(CO_{2}\right)}{100}\cdot L$$

(6) $$v\left(SO_{2}\right)=\frac{V_{m}\left(SO_{2}\right)}{\left(M\right)\left(S\right)}$$

(7) $$v_{N_{2}}=\frac{V_{m}\left(N_{2}\right)}{M\left(N_{2}\right)}\cdot N+O\frac{C_{atm}\left(N_{2}\right)}{C_{atm\;}{\left(O_{2}\right)}_{2,min}}$$

The calorific value *Q_i_* of the original water content *W* converted to the calorific value *Q_in_* at the targeted water content *W_t_* was derived using the following equation:

(8) $$Q_{in}=\frac{100-W_{t}}{100-W}\cdot \left(Q_{i}+24.42\cdot W\right)-24.42\cdot W_{t}$$

where *W* (% wt.) is the original sample’s water content and *Q_i_* is the sample’s net calorific value at the original water content, and where *W_t_* (% wt.) is the target sample’s water content and *Q_in_* is the sample’s net calorific value at the target water content.

### 2.3. Phytotoxicity Test

The toxicities of biochar extracts were evaluated on seeds of *Lepidium sativum* L., which are sensitive to the presence of toxic substances. Phytotoxicity testing was performed according to described method of Tunklova. The pyrolyzed substance was extracted into an aqueous solution using Tunklova’s outlined procedure [65]. Garden cress seed toxicity of biochar water extracts was assessed (*Lepidium sativum* L.). A Petri plate lined with one sheet of filter paper that had previously been soaked with test solution contained thirty seeds (5 mL). The control utilized was distilled water. Each testing version was prepared in five Petri dishes. The seeds were germinated for 48 h in a dark, 25 °C incubator.

The germination Index, a measure of the toxicity of biochar, was calculated using the following equation:

(9) $$GI=\frac{k_{v}\cdot l_{v}}{k_{k}\cdot l_{k}}\cdot 100\;\left(\%\right)$$

*k_v_*—the sample’s germination (%);

*k_k_*—the control’s variant germination (%);

*l_v_*—the average root length of the sample on (mm);

*l_k_*—the average root length of the control (mm).

Each sample’s root and hypocotyl lengths were also measured. If the radicle of a seed was longer than 2 mm, it was deemed to have physiologically germinated.

### 2.4. Preparation of the Biochar Extract

Methanol extracts were created in accordance with Tunklova’s instructions to measure total phenolics, flavonoids, total antioxidant activity, and tannin content [65]. In 5 mL of 70% methanol, a 0.2 g sample was weighed and added. The extract was then heated in an ultrasonic bath for 45 min to 70 °C. The samples were then centrifuged for 15 min at 2350 g. In a volumetric flask measuring 10 mL, the supernatant was kept. Once more, this extraction process was carried out. The extracts were then combined and poured into a final amount of 10 mL. Three times each of the methanol extract variations was used.

#### 2.4.1. Determination of the Total Phenolic Content

According to Singleton and Rossi [66] and Tsantili [67], the Folin–Ciocalteu method (Sigma-Aldrich, Saint Louis, MO, USA) was used to determine the total phenolic concentration. In summary, 0.7 mL of distilled water was added to a tube along with 0.25 mL of the methanol extract. After adding 0.75 mL of Folin–Ciocalteu reagent, the tube was swirled and left to stand at room temperature for 6 min. The combination also contained 0.8 mL of 7% sodium carbonate from Lachner, Neratovice, Czech Republic. The mixture was left in the dark for 90 min. A UV-Vis spectrophotometer (Evolution 210, Thermo Fisher Scientific, Waltham, MA, USA) was used to quantify absorbance in comparison with a blank at a wavelength of 760 nm. Gallic acid equivalents were used to quantify the total phenolic content on a dry basis (GAE mg g^−1^ d.b.).

#### 2.4.2. Determination of the Total Flavonoid Content

The methodology previously reported by Chang [68] using methanolic extracts was used to determine the total flavonoid content using the colorimetric method with aluminum chloride (Lachner, Neratovice, Czech Republic). The 0.2 mL methanol extracts were mixed with 0.12 mL of 5% sodium nitrate (Lachner, Neratovice, Czech Republic), 0.12 mL of 10% aluminum chloride, 0.8 mL of 1 M sodium hydroxide (Lachner, Neratovice, Czech Republic), and 0.56 mL of distilled water. The samples’ absorbances were then calculated at a wavelength of 415 nm in comparison with a control. Quercetin equivalents were used to compute the total flavonoid content (QE mg g^−1^ d.b.).

#### 2.4.3. Determination of the Total Antioxidant Activity

The phospho-molybdenum technique, as reported by Prieto [69], was used to measure the total antioxidant activity. The sample tubes underwent a 90 min incubation period at 95 °C in a water bath before being cooled to room temperature. A spectrophotometer was used to test the solution’s absorbance at 765 nm in comparison with a blank. Amounts of ascorbic acid equivalents were used to measure total antioxidant activity (AAE mg g^−1^ d.b.).

#### 2.4.4. Determination of the Tannin Content

According to Chandran and Chandran, the Folin–Ciocalteu method was used to determine the total tannin concentration [70]. To achieve a final volume of 5 mL of distilled water, 0.05 mL of the sample was combined with 3.75 mL of distilled water, 0.25 mL of Folin–Ciocalteu reagent, and 0.5 mL of 35% sodium carbonate (Lachner, Neratovice, Czech Republic). After properly stirring, the mixture was left at room temperature for 30 min. A spectrophotometer was used to measure the solution’s absorbance at 700 nm in comparison with a blank. Three duplicates of the tannin content test were run. Tannic acid equivalents were used to measure total tannin concentration. Three duplicates of the tannin content test were run. Tannic acid equivalents were used to measure the total tannin concentration (TAE mg g^−1^ d.b.). 

#### 2.4.5. Determination of Juglone Content

Only walnut shells were used for the spectrophotometric measurement of juglone concentration [71]. After samples were centrifuged at 2830× *g* for 15 min, 0.2 g of walnut shells was homogenized in 10 mL of methanol using an ultrasonic bath. Once more, this extraction process was carried out. The extracts were then combined and poured into a final amount of 10 mL. At a wavelength of 410 nm, absorbance samples were measured on a spectrophotometer. Pure methanol comprised the blank sample. The juglone content was described as mg g^−1^ d.b. endocarp.

#### 2.4.6. Chemical Analysis of Materials

The chemical analysis was performed according to the TAPPI Test methods [72]. The samples were first analyzed for the extractive organic part which was determined according to normal procedures (TAPPI T 280 pm-99). Using the Soxhlet equipment, extraction was carried out with ethanol and toluene in a 7:3 ratio as the extraction solvent. The Seifert technique was used to determine the amount of cellulose (Wright and Wallis 1998) [73] and Klason lignin according to normal procedures (TAPPI T 222 om-02) using sulfuric acid. The holocellulose was determined by the method of [74]. The approximate hemicellulose content was computed using the amounts of holocellulose and cellulose.

### 2.5. Statistical Analysis

Statistical processing of the results was performed using STATISTICA 12.0 CZ (StatSorft, Tusla, OK, USA) program. All data were tested using one-way ANOVA followed by post-hoc comparison with Tukey’s (least significant difference) test (*p* < 0.05).

## 3. Results and Discussion

### 3.1. Proximate and Ultimate Analysis

#### 3.1.1. Proximate and Ultimate Analysis of Walnut Shells

The walnut shells were characterized by a high ash content of 2.61% wt. in the WAL0 sample, which was the highest among the shells analyzed; similarly, high ash content was also analyzed for Miscanthus sinensis [75]. The share of carbon and oxygen was 48.77% wt. and 42.28% wt., respectively, values similar to, for instance, tea waste [65]. 

In the case of the pyrolyzed material, the proportion of carbon increased significantly with increasing temperature up to 84.66% wt. for WAL550. Similarly, the net calorific value of the material increased from 18.66 MJ kg^−1^ for WAL0 up to 30.55 MJ kg^−1^ for WAL550. 

Like the other materials tested, the oxygen and hydrogen content decreased with increasing pyrolysis temperature, most significantly for oxygen to 1.74% wt. in WAL550. The ash content of walnut shells was high, increasing from 2.48% wt. for WAL0 up to 10.12% wt. in WAL550. Such a high content of ash may be problematic for combustion chambers, as was determined for wheat straws [76], or other lignocellulosic biomasses such as tree cones [77]. For more detail, see Table 2. Among other research, we can mention the research of Zhu [48], according to whom walnut shells are used as a typical biomass waste to prepare biochar and bio-oil by pyrolysis.

#### 3.1.2. Proximate and Ultimate Analysis of Pistachio Shells

Pistachio shells contained a high share of oxygen, 43.65 wt.%, which was the highest value of the studied primary materials and similar to that of wood [78]. 

The carbon content of pistachio shells was 48.72% wt. and it increased with pyrolysis temperature up to 89.03% wt. for PIST550. In the sample PIST550, the carbon content of pistachio shells was slightly higher than that of walnut shells, although the initial carbon content of the original sample was almost identical for both materials, i.e., slightly below 49% wt. The net calorific value of pistachio varied from 17.58 MJ kg^−1^ in PIST0 up to 31.35 MJ kg^−1^ in PIST550. 

The proportions of oxygen and hydrogen decreased with the pyrolysis severity and the nitrogen level increased slightly. The share of ash increased from 1.09% wt. of PIST0 up to 4.84% wt. at PIST550 (see Table 3).

#### 3.1.3. Proximate and Ultimate Analysis of Peanut Shells

Peanut shells contained a high carbon content of 53.15% wt. and a relatively low ash content of 1.87% wt. However, they contained a high nitrogen content of 1.47% wt., the highest among the samples analyzed. The elemental analysis results of the peanut shells were very similar to those of coffee grounds [79].

The carbon share increased with pyrolysis temperature to 84.00% wt. for PEAN550. Among all the nutshell samples, the carbon share increased the least with increased temperature. Similarly, the net calorific value of the material increased from 19.93 MJ kg^−1^ for PEAN0 to 28.78 MJ kg^−1^ for PEAN550. Peanut shells had the highest net calorific value of the original materials; however, during pyrolysis treatment they recorded the slowest increase.

The residual oxygen value decreased with increasing temperature compared with the original material and amounted to 5.09% wt. for PEAN550, the highest of the shells studied (i.e., 1.74% wt. for WAL550, 2.72% wt. for PIST550). The ash content increased with temperature from 1.87% wt. for PEAN0 to 6.30% wt. for PEAN550 (see Table 4). 

### 3.2. Stoichiometric Analysis

Stoichiometric analysis is the basis of thermal calculations and helps to estimate the combustion behavior of any material. Theoretical and real mass and volumetric amounts were calculated for several variables, e.g., the amount of air for combustion and the amount of dry flue gas. The emission values were converted to dry flue gas at a temperature of 273.15 K, a pressure of 101.325 kPa, and a reference oxygen content of 10% according to the requirements of the EU legislation for stationary combustion sources for solid fuels. Additionally, calculations of fuel throughputs were performed for combustion devices with nominal heat inputs from 10 to 300 kW, which could serve as heat sources for hot water central heating systems. 

For walnut shells, there was a significant increase in the amount of combustion air required for perfect combustion with increasing pyrolysis temperature between WAL250 and WAL300. This increase was at a lower level compared with coffee waste [79], tea waste [65], or by-products such as grape pomace [80], for which this increase usually occurs above 350 °C. A similar increase between WAL250 and WAL300 was also valid for dry flue gas concentrations with the mass amount of CO_2_ and N_2_. The necessary fuel throughput of walnut shells for a 300 kW source decreased with increasing pyrolysis temperature because of the increasing proportion of combustible components, hence increasing the calorific value from 64.30 kg h^−1^ of WAL0 to only 39.27 kg h^−1^ of WAL550. By reducing the necessary mass throughput, there are also substantial savings in the handling of the material. In terms of flue gas concentrations and calorific value, a temperature of 300 °C can be recommended for walnut shells (see Table 5).

Pistachio shells followed a similar pattern to walnut shells, i.e., between PIST250 and PIST300, but not quite as extreme. At the mentioned level, the theoretical amount of air for perfect combustion and the mass amount of dry flue gas increased with pyrolysis severity. From the aspect of flue gas concentration and calorific value of the material, pyrolysis of pistachio shells can also be recommended at 300 °C (see Table 6).

Peanut shells showed an increase in their requirement for air for perfect combustion in the diffusion areas during pyrolysis as low as PEAN300. As the pyrolysis temperature of the fuel treatment increased, the mass amount of dry flue gas increased. The amount of CO_2_ was balanced for all peanut shell samples. Therefore, a maximum temperature of 550 °C can be recommended for peanut shells, which achieved the highest calorific value and, at the same time, had an acceptable flue gas production (see Table 7).

To determine the energy consumption for the preparation of biochar of these waste samples, the heat capacity of biomass can be used as a guide. The magnitude of heat capacity (J kg^−1^ K^−1^) compared with different biomasses is significant [81]. Values of about 1000 J kg^−1^ K^−1^ have been obtained on biochar regardless of the pyrolysis temperature [82]. The average value of the heat capacity of the used samples was determined by linear correlation based on the measured values for lignocellulosic material [82], and its average value was about 1095 J kg^−1^ K^−1^. The energy consumption was determined by the required pyrolysis temperature, which is given in kJ kg^−1^. For each pyrolysis level, the energy consumption was approximately 274 kJ kg^−1^ for 250 °C, 329 kJ kg^−1^ for 300 °C, 383 kJ kg^−1^ for 350 °C, 493 kJ kg^−1^ for 450 °C, and 602 kJ kg^−1^ for 550 °C. Further calculations should include any heat losses that depend on the actual pyrolysis conditions.

### 3.3. Phytotoxicity Test

**Figure 1:**
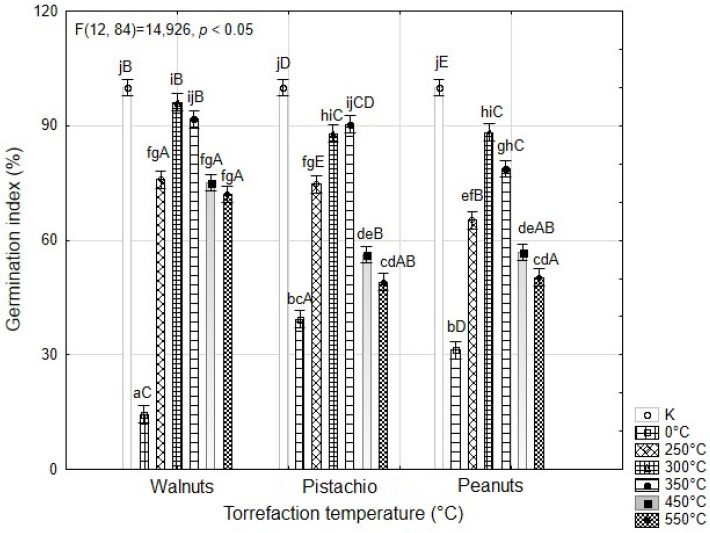
The phytotoxicity of the aqueous extracts of walnut, pistachio, and peanut shells on the germination of *Lepidium sativum* L. seeds after 48 h. Data are expressed as means of five independent bioassays (five replicates for each concentration (aqueous extracts) per bioassay) ± standard error. Different lowercase letters (a–j) indicate significant differences among the seven torrefaction temperatures in a specific aqueous extracts of nut shells (ANOVA, Tukey’s test, *p* < 0.05); Different capital letters (A–E) indicate significant differences in peanuts, walnuts and pistachios at different torrefaction temperatures (ANOVA, Tukey’s test, *p* < 0.05).

As well as ground coffee and waste from tea production [65,79], nutshells could be suitable for soil applications. Figure 1 compares the germination indices of the control samples with the analyzed peanut, walnut, and pistachio shell samples before and after pyrolysis. The germination indexes of the original samples of peanut, walnut, and pistachio shells were 31%, 14%, and 39%, respectively. Walnut shells showed the strongest phytotoxic effect. According to Matok [83], walnut shells contain substances such as polyphenols, which include flavonoids, phenolics and tannins. These substances can have a negative effect on seed germination. Another substance that may have a negative effect on seed germination is a phenol juglone. According to Cosmulescu [84], juglone can be found in all parts of the plant but is usually present in higher concentrations in the buds, nut hulls, and roots. Lesser amounts are found in the leaves, stems, and shells [85]. As shown in Figure 1, the phytotoxicity decreased with increasing pyrolysis temperature. The highest germination index was found for biochar from walnut shells at the level of 92% for WAL350. The germination of biochar from walnuts was also studied by Uslu [86]. Their results are different from those presented herein, probably because they studied germination on different plant species. For peanut and pistachio samples at temperatures of 550 °C, biochar should no longer be applied to the soil since the germination index of these samples is less than 50%. Deenik [87] found that biochar water extracts from macadamia nut shells (430 °C) had a strong phytotoxic effect on the germination of radish and corn seeds. Additionally, Buss and Mašek [88] found that extracts of biochar produced from pellets at 550 °C induced heavy phytotoxicity in germination seeds of garden cress. Very similar results for walnut, almond, and peanut shells were also determined by Fetjah [89]. According to Solaiman [90], the germination index is significantly affected by biochar concentration, material, and the chemical composition of the material, as well as pyrolysis temperature and duration.

### 3.4. Chemical Analysis

Secondary metabolites such as polyphenols, flavonoids, tannins, and others are widespread in nut shells. Total phenolics (TPC), flavonoids (TFC), total antioxidant activity (TAA), and tannic activity (TAE) are shown in Table 8. The highest phenolic content was found in walnut and pistachio shells pyrolyzed at 250 °C. The values of total phenolic compounds ranged from 0.07 mg g^−1^ to 9.3 mg g^−1^ for peanuts. In peanut shells, the highest measured value was for the non-pyrolyzed material. The published data on phenolic content in walnut shells extracts vary greatly. As with the phenolic content, the highest flavonoid content was found in walnut and pistachio shells pyrolyzed at 250 °C. Yang [91] measured a higher content of phenolic compounds and flavonoids compared with our results. The total phenolic content of the samples was very close to the results of Jalili [92] and Han [93]. These results are different from those reported before by Queirós [94]. According to Soto-Maldonado [95], the differences between TFC and TPC are mainly due to the polarity of the solvent and the solubility of the sample in the extract or in the solvent. The total antioxidant activity of nutshells ranged from 0.07 mg g^−1^ to 39.49 mg g^−1^ for walnut shells, from 0.12 mg g^−1^ to 47.75 mg g^−1^ for pistachio shells, and from 0.13 mg g^−1^ to 25.63 mg g^−1^ for peanut shells. The total tannin content of the nutshells ranged from 0.41 mg g^−1^ to 13.49 mg g^−1^ for walnut shells, from 0.36 mg g^−1^ to 15.56 mg g^−1^ for pistachio shells, and from 0.38 mg g^−1^ to 9.78 mg g^−1^ for peanut shells. Dolatabadi [96] reported the content of condensed tannins in the methanol extract of almond shells and walnuts to be 60.1 and 34.6 mg g^−1^, which is more comparable to the results presented herein (Table 8).

The juglone content of the walnut shells significantly varied with pyrolysis temperature, which was in the range of 0.29 mg g^−1^ to 0.99 mg g^−1^ (Table 9). Juglone is characterized by a strong phytotoxic effect. The toxic effect was also evident from our results. The highest juglone content was found in the non-pyrolyzed walnut sample. Soto-Maldonado [95] detected that juglone was only present in the samples of walnut hulls, as opposed to walnut shells, in which juglone could not be detected. However, there are very few studies on the determination of juglone content in walnut shells.

The main chemical constituents of walnut, pistachio, and peanuts shells are cellulose, lignin, hemicellulose, and soluble carbohydrates, which are similar to those of wood materials. In comparison with walnut and pistachio shells, peanut shells had lower proportions of hemicellulose. Peanut shells have a complex fibrous structure [97]. Table 10 shows the average chemical composition of the investigated walnut, pistachio, and peanut shell samples before and after pyrolysis. The chemical composition in nut shells depends on the climate, plant species, and genotypes. For this reason, chemical composition values in similar slow pyrolysis studies may differ. According to Waters [98], hemicellulose, cellulose, and lignin decompose at different temperature ranges. In general, hemicellulose decomposes at lower temperatures (220–315 °C). Cellulose is also more resistant to high temperatures than hemicellulose. Cellulose depolymerization occurs in the temperature range 300–400 °C. At this temperature, volatiles are also rapidly decomposed. Lignin thermally degrades over a wide temperature range (150–900 °C) and is thus the most stable component of the material [99]. It is known that the chemical composition of nutshells depends on the part of the plant, the method of processing and drying, pyrolysis temperature, climate, and even the location of cultivation as well as many other factors. Table 10 shows that the number of extractives and holocellulose both decrease with increasing pyrolysis temperature. At the same time, it is possible to observe an increase in cellulose and lignin content for each sample. Klason’s method, chosen for lignin determination, yielded almost similar results (29.3% wt. lignin for walnut shells, 28.1% wt. lignin for almond shells, and 39.4% wt. lignin for pine nut shells) as those in the study by Queirós [94].

## 4. Conclusions

The results from the analyses showed that the use of waste from nut production is energetically justified not only in terms of reducing emission concentrations, but especially in enhancing the fuel quality of such waste. An important aspect of these by-products is that they can be used as energetically valuable fuels in a circular economy, leading to cost savings, especially in the reduced fuel weight to be transported, thus reducing handling costs. Results from the model simulations of a heating plant clearly show the process parameters for optimal use of these wastes as biochar.

Walnut shells contain a high amount of ash, increasing with the pyrolysis temperature up to 10.12% wt., simultaneously increasing the calorific value up to 30.55 MJ kg^−1^. There was a spike in the mass concentration of dry flue gas between the pyrolysis temperature of 250 °C and 300 °C, which was a determining factor for setting the ideal pyrolysis temperature of walnut shells to 300 °C for the purpose of their use as fuels, considering the high ash content, i.e., to adapt the combustion process accordingly. To apply walnut shells as a soil amendment, the recommended pyrolysis temperature was 300 °C, which achieved the lowest phytotoxicity level.

Pistachio shells contained the lowest proportion of ash, and showed a similar increase in calorific value with increasing pyrolysis temperature and a similar spike in emission values between 250 and 300 °C. Thus, for pistachio shells, a pyrolysis temperature of 300 °C is also suitable for combustion purposes. For application as a soil amendment, the sample pyrolyzed at 350 °C had the lowest phytotoxicity.

For peanut shells, there was an increase in production of combustion gases and CO_2_ emissions from the lowest pyrolysis temperature up to 300 °C, above which the values became stable. Thus, for combustion purposes, the possibility of torrefying peanut shells at a maximum measured temperature of 550 °C can be considered to achieve the highest calorific value of the material. As a soil amendment, peanut shells performed best at 300 °C pyrolysis, as with the other materials.

## Figures and Tables

**Table 1:** Samples listed by pyrolysis temperature.

List of Samples	Walnut	Pistachio	Peanut
Non-pyrolyzed	WAL0	PIST0	PEAN0
250 °C	WAL250	PIST250	PEAN250
300 °C	WAL300	PIST300	PEAN300
350 °C	WAL350	PIST350	PEAN350
450 °C	WAL450	PIST450	PEAN450
550 °C	WAL550	PIST550	PEAN550

**Table 2:** Analysis of walnut shells’ proximal and ultimate dry values; different letters indicate significant differences between samples based on Tukey’s test (*p* < 0.05).

	Ash (% wt.)	Carbon (% wt.)	Hydrogen (% wt.)	Nitrogen (% wt.)	Sulfur (% wt.)	Oxygen (% wt.)	Net Calorific Value (MJ kg^−1^)
WAL0	2.61 ± 0.14 ^a^	48.77 ± 0.72 ^b^	5.80 ± 0.10 ^a^	0.52 ± 0.02 ^a^	0.012 ± 0.002	42.28	18.66
WAL250	3.25 ± 0.19 ^a^	55.57 ± 0.8 ^c^	5.71 ± 0.08 ^a^	0.49 ± 0.03 ^a^	0.003 ± 0.001	34.98	20.14
WAL300	4.59 ± 1.33 ^ab^	67.13 ± 0.25 ^d^	5.34 ± 0.02 ^e^	0.57 ± 0.02 ^a^	0.005 ± 0.001	22.37	25.21
WAL350	6.81 ± 0.4 ^bc^	75.47 ± 1.48 ^e^	4.24 ± 0.01 ^d^	0.70 ± 0.00 ^b^	0.008 ± 0.001	12.77	26.13
WAL450	8.96 ± 0.8 ^cd^	82.81 ± 0.45 ^a^	3.41 ± 0.01 ^c^	0.78 ± 01.01 ^bc^	0.007 ± 0.001	4.04	28.85
WAL550	10.12 ± 0.2 ^d^	84.66 ± 1.9 ^a^	2.66 ± 0.01 ^b^	0.82 ± 0.00 ^c^	0.005 ± 0.001	1.74	30.55

**Table 3:** Pistachio shell proximal and ultimate analysis, with dry values reported; different letters indicate significant differences between samples based on Tukey’s test (*p* < 0.05).

	Ash (% wt.)	Carbon (% wt.)	Hydrogen (% wt.)	Nitrogen (% wt.)	Sulphur (% wt.)	Oxygen (% wt.)	Net Calorific Value (MJ kg^−1^)
PIST0	1.09 ± 0.07 ^a^	48.72 ± 0.08 ^a^	6.14 ± 0.02 ^f^	0.40 ± 0.02 ^a^	0.004 ± 0.001	43.65	17.58
PIST250	1.48 ± 0.11 ^ab^	54.96 ± 0.08 ^b^	5.85 ± 0.02 ^e^	0.43 ± 0.01 ^ab^	0.012 ± 0.001	37.26	20.17
PIST300	2.38 ± 0.18 ^bc^	66.83 ± 0.21 ^c^	5.26 ± 0.02 ^d^	0.49 ± 0.01 ^b^	0.009 ± 0.001	25.04	25.06
PIST350	2.80 ± 0.19 ^c^	77.11 ± 0.27 ^d^	4.30 ± 0.01 ^c^	0.57 ± 0.00 ^c^	0.012 ± 0.001	15.21	27.93
PIST450	4.33 ± 0.34 ^d^	83.88 ± 0.06 ^e^	3.44 ± 0.00 ^b^	0.62 ± 0.00 ^c^	0.018 ± 0.002	7.71	29.71
PIST550	4.84 ± 0.35 ^d^	89.03 ± 0.19 ^f^	2.69 ± 0.00 ^a^	0.72 ± 0.02 ^d^	0.011 ± 0.001	2.72	31.35

**Table 4:** Peanut shell proximal and ultimate analysis, with values reported on a dry basis; different letters indicate significant differences between samples based on Tukey’s test (*p* < 0.05).

	Ash (% wt.)	Carbon (% wt.)	Hydrogen (% wt.)	Nitrogen (% wt.)	Sulphur (% wt.)	Oxygen (% wt.)	Net Calorific Value (MJ kg^−1^)
PEAN0	1.87 ± 0.02 ^a^	53.15 ± 0.14 ^a^	6.17 ± 0.03 ^a^	1.47 ± 0.04 ^b^	0.064 ± 0.005	37.27	19.93
PEAN250	2.15 ± 0.05 ^b^	56.87 ± 0.06 ^b^	6.07 ± 0.04 ^a^	1.59 ± 0.02 ^bcd^	0.037 ± 0.003	33.28	20.61
PEAN300	2.99 ± 0.01 ^c^	64.31 ± 0.1 ^c^	5.72 ± 0.03 ^e^	1.69 ± 0.04 ^cd^	0.044 ± 0.004	25.24	24.33
PEAN350	4.3 ± 0.1 ^d^	73.65 ± 0.17 ^d^	4.49 ± 0.01 ^d^	1.98 ± 0.05 ^a^	0.035 ± 0.004	15.55	26.38
PEAN450	6.04 ± 0.04 ^e^	78.68 ± 0.31 ^e^	3.31 ± 0.02 ^c^	1.93 ± 0.04 ^a^	0.040 ± 0.005	10.00	27.35
PEAN550	6.30 ± 0.06 ^f^	84.00 ± 0.18 ^f^	2.60 ± 0.02 ^b^	1.96 ± 0.04 ^a^	0.042 ± 0.004	5.09	28.78

**Table 5:** Combustion characteristics of walnut shells and their biochar.

	WAL0	WAL250	WAL300	WAL350	WAL450	WAL550
Mass flow of fuel for a heat output of 10 kW (kg h^−1^)	2.14	1.99	1.59	1.53	1.39	1.31
Mass flow of fuel for a heat output 300 kW (kg h^−1^)	64.30	59.58	47.61	45.92	41.60	39.27
Theoretical amount of O_2_ for complete combustion (kg kg^−1^)	1.34	1.59	1.99	2.22	2.44	2.45
Theoretical amount of air for complete combustion (kg kg^−1^)	5.78	6.85	8.59	9.59	10.52	10.57
Real mass amount of air (*n* = 1.91) (kg kg^−1^)	11.37	13.38	16.70	18.31	20.09	20.19
Theoretical mass amount of dry flue gas (kg kg^−1^)	6.16	7.21	8.95	10,1	10.98	11.09
Real mass amount of dry flue gas (*n* = 1.91) (kg kg^−1^)	11.37	13.38	16,70	18.65	20.47	20.62
Theoretical mass amount of CO_2_ (kg kg^−1^)	1.79	2.04	2.47	2.77	3.04	3.11
Theoretical mass amount of H_2_O (kg kg^−1^)	0.75	0.79	0.82	0.76	0.73	0.66
Theoretical mass amount of N_2_ (kg kg^−1^)	4.37	5.17	6.49	7.24	7.95	7.99
Theoretical mass amount of CO_2_ (% wt.)	29.06	28.23	27.48	27.63	23.38	23.71

**Table 6:** Combustion characteristics of pistachio shells and their biochar.

Pistachio Shells	PIST0	PIST250	PIST300	PIST350	PIST450	PIST550
Mass flow of fuel for a heat output of 10 kW (kg h^−1^)	2.26	1.98	1.60	1.43	1.35	1.28
Mass flow of fuel for a heat output 300 kW (kg h^−1^)	68.26	59.51	47.88	42.97	40.39	38.27
Theoretical amount of O_2_ for complete combustion (kg kg^−1^)	1.35	1.56	1.95	2.25	2.44	2.56
Theoretical amount of air for complete combustion (kg kg^−1^)	5.64	6.73	8.41	9.69	10.50	11.04
Real mass amount of air (*n* = 1.91) (kg kg^−1^)	11.15	12.85	16.07	18.51	20.05	21.09
Theoretical mass amount of dry flue gas (kg kg^−1^)	6.19	7.10	8.81	10.15	11.00	11.61
Real mass amount of dry flue gas (*n* = 1.91) (kg kg^−1^)	11.45	13.16	16.39	18.88	20.47	21.56
Theoretical mass amount of CO_2_ (kg kg^−1^)	1.79	2.02	2.45	2.83	3.08	3.27
Theoretical mass amount of H_2_O (kg kg^−1^)	0.79	0.80	0.81	0.77	0.73	0.68
Theoretical mass amount of N_2_ (kg kg^−1^)	4.41	5.08	6.36	7.32	7.93	8.34
Theoretical mass amount of CO_2_ (% wt.)	28.84	28.39	27.83	27.86	27.95	28.13

**Table 7:** Combustion characteristics of peanut shells and their biochar.

Peanut Shells	PEAN0	PEAN250	PEAN300	PEAN350	PEAN450	PEAN550
Mass flow of fuel for a heat output of 10 kW (kg h^−1^)	2.01	1.94	1.64	1.52	1.46	1.39
Mass flow of fuel for a heat output 300 kW (kg h^−1^)	60.21	58.24	49.33	45.48	43.88	41.69
Theoretical amount of O_2_ for complete combustion (kg kg^−1^)	1.54	1.67	1.92	2.17	2.26	2.40
Theoretical amount of air for complete combustion (kg kg^−1^)	6.63	7.20	8.28	9.35	9.76	10.33
Real mass amount of air (*n* = 1.91) (kg kg^−1^)	12.67	13.75	15.81	17.85	18.63	19.74
Theoretical mass amount of dry flue gas (kg kg^−1^)	6.97	7.53	8.62	9.77	10.27	10.90
Real mass amount of dry flue gas (*n* = 1.91) (kg kg^−1^)	12.95	14.02	16.08	18.20	19.06	20.21
Theoretical mass amount of CO_2_ (kg kg^−1^)	1.95	2.09	2.60	2.70	2.89	3.08
Theoretical mass amount of H_2_O (kg kg^−1^)	0.82	0.83	0.85	0.78	0.69	0.65
Theoretical mass amount of N_2_ (kg kg^−1^)	5.02	5.45	6.26	7.07	7.38	7.82
Theoretical mass amount of CO_2_ (% wt.)	27.96	27.68	27.35	27.63	28.10	28.26

**Table 8:** Total phenolic (TPC), flavonoid (TFC), and antioxidant activity (TAA), and total tannin content (TAE) of walnut, pistachio, and peanut shell methanol extracts. The values are mean standard deviation (*n* = 3). Different letters (^a–f^) indicate Tukey’s test showed significant differences (*p* < 0.05).

Sample	TPC (mg g^−1^)	TFC (mg g^−1^)	TAA (mg g^−1^)	TAE (mg g^−1^)
WAL0	3.45 ± 0.12 ^c^	6.65 ± 0.55 ^c^	13.89 ± 0.83 ^c^	4.32 ± 0.1 ^d^
WAL250	9.85 ± 0.08 ^d^	15.48 ± 0.88 ^d^	39.49 ± 1.51 ^d^	13.49 ± 0.16 ^e^
WAL300	2.12 ± 0.06 ^b^	3.75 ± 0.17 ^b^	5.73 ± 0.18 ^b^	3.19 ± 0.26 ^c^
WAL350	0.16 ± 0.01 ^a^	1.4 ± 0.04 ^a^	0.89 ± 0.04 ^a^	1.62 ± 0.07 ^b^
WAL450	0.07 ± 0.01 ^a^	0.81 ± 0.04 ^a^	0.16 ± 0.00 ^a^	0.61 ± 0.23 ^a^
WAL550	0.04 ± 0.00 ^a^	0.62 ± 0.01 ^a^	0.07 ± 0.00 ^a^	0.41 ± 0.03 ^a^
PIST0	3.42 ± 0.10 ^c^	13.08 ± 0.10 ^c^	11.02 ± 0.01 ^c^	3.03 ± 0.08 ^c^
PIST250	11.77 ± 0.40 ^d^	20.46 ± 1.17 ^d^	47.75 ± 1.92 ^d^	15.56 ± 0.38 ^e^
PIST300	1.94 ± 0.05 ^b^	6.15 ± 0.02 ^b^	4.37 ± 0.06 ^b^	4 ± 0.06 ^d^
PIST350	0.13 ± 0.01 ^a^	1.10 ± 0.03 ^a^	1.14 ±0.07 ^ab^	1.38 ± 0.08 ^b^
PIST450	0.05 ± 0.00 ^a^	1.08 ± 0.04 ^a^	0.17 ± 0.01 ^a^	0.64 ± 0.06 ^ab^
PIST550	0.04 ± 0.00 ^a^	0.92 ± 0.04 ^a^	0.12 ± 0.01 ^a^	0.36 ± 0.02 ^a^
PEAN0	9.30 ± 0.13 ^e^	12.53 ± 0.11 ^e^	25.63 ± 0.53 ^d^	9.78 ± 0.03 ^f^
PEAN250	6.28 ± 0.21 ^d^	9.73 ± 0.21 ^d^	10.42 ± 0.57 ^b^	7.46 ± 0.13 ^e^
PEAN300	3.48 ± 0.02 ^c^	6.00 ± 0.04 ^c^	9.85 ± 0.51 ^b^	4.41 ± 0.14 ^d^
PEAN350	0.66 ± 0.02 ^b^	2.29 ± 0.13 ^ab^	2.32 ± 0.06 ^b^	1.57 ± 0.06 ^c^
PEAN450	0.14 ± 0.02 ^a^	1.08 ± 0.02 ^a^	0.42 ± 0.01 ^a^	0.81 ± 0.04 ^b^
PEAN550	0.07 ± 0.01 ^a^	0.98 ± 0.03 ^a^	0.13 ± 0.01 ^a^	0.38 ± 0.01 ^a^

**Table 9:** The juglone content of a walnut shell methanol extract. Values are standard deviations (*n* = 3). Different letters (^a–e^) indicate that Tukey’s test showed significant differences (*p* < 0.05).

Sample	Junglone (mg g^−1^)
non-pyrolyzed	0.99 ± 0.00 ^e^
WAL250	0.88 ± 0.04 ^d^
WAL300	0.8 ± 0.00 ^a^
WAL350	0.77 ± 0.00 ^a^
WAL450	0.52 ± 0.00 ^c^
WAL550	0.29 ± 0.00 ^b^

**Table 10:** Chemical composition of peanut, walnut, and pistachio shells (% wt. of oven-dried samples). Values in parentheses show the measurement error.

Samples	Extractives	Holocellulose (wt. %)	Cellulose (wt. %)	Hemicellulose (wt. %)	Lignin (wt. %)
WAL0	4.27 ± 0.003	74.49 ± 0.074	3.28 ± 0.003	71.21 ± 0.071	18.38 ± 0.018
WAL250	0.10 ± 0.012	73.44 ± 8.372	6.38 ± 0.727	67.06 ± 7.645	21.40 ± 2.439
WAL300	0.46 ± 0.000	68.29 ± 0.007	10.55 ± 0.001	57.73 ± 0.006	24.29 ± 0.002
WAL350	0.31 ± 0.006	58.33 ± 1.143	10.05 ± 0.197	48.27 ± 0.946	27.18 ± 0.532
WAL450	0.06 ± 0.005	54.14 ± 4.250	12.16 ± 0.954	41.98 ± 3.296	28.83 ± 2.262
WAL550	0.02 ± 0.004	46.84 ± 10.248	10.69 ± 2.339	36.15 ± 7.909	36.66 ± 8.021
PIST0	1.10 ± 0.001	82.10 ± 0.049	4.84 ± 0.003	77.26 ± 0.046	8.08 ± 0.005
PIST250	4.52 ± 0.565	67.98 ± 8.490	8.00 ± 1000	59.96 ± 7.489	18.06 ± 2.256
PIST300	1.35 ± 0.114	63.91 ± 5.388	11.36 ± 0.958	52.55 ± 4.430	24.33 ± 2.051
PIST350	1.23 ± 0.026	48.38 ± 1.021	14.11 ± 0.298	34.25 ± 0.723	31.62 ± 0.667
PIST450	1.00 ± 0.012	42.80 ± 0.458	14.22 ± 0.152	28.58 ± 0.306	31.96 ± 0.342
PIST550	1.19 ± 0.073	38.78 ± 2.393	13.54 ± 0.836	25.23 ± 1.557	32.27 ± 1.991
PEAN0	5.94 ± 0.017	67.36 ± 0.202	6.77 ± 0.020	60.60 ± 0.181	20.52 ± 0.062
PEAN250	8.01 ± 0.074	61.49 ± 0.572	8.78 ± 0.081	52.71 ± 0.490	21.51 ± 0.200
PEAN300	6.42 ± 0.667	57.27 ± 5.945	10.77 ± 1.118	46.50 ± 4.827	24.83 ± 2.578
PEAN350	1.48 ± 0.022	54.70 ± 0.815	13.74 ± 0.204	40.96 ± 0.610	28.57 ± 0.426
PEAN450	0.27 ± 0.006	50.30 ± 1.066	13.74 ± 0.291	36.56 ± 0.775	28.53 ± 0.604
PEAN550	0.20 ± 0.033	42.18 ± 4.906	12.67 ± 1.473	29.52 ± 3.433	28.34 ± 3.294

## Data Availability

The data not directly presented in the article will be made available on request.
